# Risk of psychological distress by decrease in economic activity, gender, and age due to COVID-19: A multinational study

**DOI:** 10.3389/fpubh.2023.1056768

**Published:** 2023-05-10

**Authors:** Minji Kim, Byungyoon Yun, Juho Sim, Ara Cho, Juyeon Oh, Jooyoung Kim, Kowit Nambunmee, Laura S. Rozek, Jin-Ha Yoon

**Affiliations:** ^1^Yonsei University College of Medicine, Seoul, Republic of Korea; ^2^Department of Preventive Medicine, Yonsei University College of Medicine, Seoul, Republic of Korea; ^3^Department of Public Health, Graduate School, Yonsei University, Seoul, Republic of Korea; ^4^Catholic University of Korea College of Medicine, Seoul, Republic of Korea; ^5^School of Health Science, Mae Fah Luang University, Chiang Rai, Thailand; ^6^Urban Safety Innovation Research Group (USIR), Mae Fah Luang University, Chiang Rai, Thailand; ^7^Georgetown Lombardi Comprehensive Cancer Center, Georgetown University, Washington, DC, United States; ^8^The Institute for Occupational Health, Yonsei University College of Medicine, Seoul, Republic of Korea

**Keywords:** COVID-19, job loss and unemployment, psychological distress, multinational survey, human development index

## Abstract

**Introduction:**

Coronavirus disease (COVID-19) is an infectious disease caused by the SARS-CoV-2-virus. COVID-19 has officially been declared as the latest in the list of pandemics by WHO at the start of 2020. This study investigates the associations among decrease in economic activity, gender, age, and psychological distress during the COVID-19 pandemic considering the economic status and education level of countries using multinational surveys.

**Methods:**

Online self-report questionnaires were administered in 15 countries which were spontaneously participate to 14,243 respondents in August 2020. Prevalence of decrease in economic activity and psychological distress was stratified by age, gender, education level, and Human Development Index (HDI). With 7,090 of female (49.8%), mean age 40.67, 5,734 (12.75%) lost their job and 5,734 (40.26%) suffered from psychological distress.

**Results:**

Associations among psychological distress and economic status, age, and gender was assessed using multivariate logistic regression, adjusted for country and education as random effects of the mixed model. We then measured the associations between HDI and age using multivariate logistic regression. Women had a higher prevalence of psychological distress than men with 1.067 Odds ratio, and younger age was significantly associated with decrease in economic activity for 0.998 for age increasing. Moreover, countries with lower HDI showed a higher prevalence of decrease in economic activity, especially at lower education levels.

**Discussion:**

Psychological distress due to COVID-19 revealed a significant association with decrease in economic activity, women, and younger age. While the proportion of decrease in economic activity population was different for each country, the degree of association of the individual factors was the same. Our findings are relevant, as women in high HDI countries and low education level in lower HDI countries are considered vulnerable. Policies and guidelines for both financial aid and psychological intervention are recommended.

## 1. Introduction

Zoonotic diseases are increasing at an alarming rate in nowadays. These various issues from such disease are newly emerging, and there are new diseases that are re-emerging (1). Especially COVID-19, which has officially been declared as the latest in the list of pandemics by WHO at the start of 2020, has caused over 6.80 million deaths as of January 2023 (2–4). While the first wave of COVID-19 made a huge impact, various variants have since changed the face. In particular, the Omicron version has been the only one in circulation for quite some time, and subvariants mutations have also been emerged (5). There are recent researches that SARS-COV-2 genetic markers in wastewater is useful to identify high-prevalence location of COVID-19 infection, which might reduce the burden of the public health system during COVID-19 pandemics (6, 7).

The COVID-19 also affects psychological distress in several ways, including fear and uncertainty, social isolation, financial stress, health anxiety, and trauma. Based on Cognitive Appraisal Theory, the pandemic has been a major stressor for many people and individuals’ cognitive appraisals of the situation can play a significant role in determining their emotional and behavioral responses (8). For example, individuals who view the pandemic as a threat to their health or economic status may experience greater levels of anxiety or fear. Moreover, biopsychosocial model suggest that the pandemic has had a wide range of impacts on individuals’ physical health, psychological well-being, and social interactions (9). For example, individuals who experience physical symptoms of COVID may also experience anxiety or depression, and social distancing measures may limit social support and exacerbate feelings of isolation.

Psychological distress refers to increased depressive symptom of anxiety symptom during pandemic. Psychological distress symptoms, of all levels, are associated with an elevated risk of all-cause and cardiovascular-specific mortality (10). Also, some research shows psychological distress is associated with suicide mortality. Moreover, such psychological distress cause significant economic burden (11, 12). Especially this study focused on the part of pandemic which has had a significant impact on the global economy, and cause numerous job loss or financial hardship.

To prevent the worldwide spread of COVID-19, social distancing policies were chosen as non-pharmaceutical interventions (13). Recession due to COVID-19 consequently led to diminishing employment status in various industries such as service and manufacturing sectors; the leisure and hospitality industries have been impacted the hardest (14). Specifically, the instability of the global economy caused a massive loss of employment, which reached an estimated 20 million jobs by April 6, 2020, far more than jobs lost during the entire Great Recession (15). Considering that economic activity is an important factor to maintain quality of life and job loss is one of the most crucial issues in the occupational health area (16, 17), early retirement and phobia due to the pandemic can aggravate the mental health of workers (18).

Financial stress can contribute to anxiety and depression. Furthermore, these people may become more susceptible to mental health impairments, including suicidality (16, 18). Moreover, individuals who are facing unemployment go through significant stress not only with job loss but also during the job search (18). This study draws attention to the psychological trauma that can result from decrease in economic activity and job search, and motivates psychologists to consider issues of work-life spillover in the aftermath of the pandemic.

Importantly, the impact of a recession does not affect all individuals and all countries equally (19). Gender, age, employment, income, education level, and social relationships are individual factors that have a bearing on resilience (19). Socio-economic factors can also influence these effects. Analysis of the policies implemented in some countries during economic crises reveals a link between these policies and their impact on the mental health of the population (20, 21). In this respect, vulnerable groups—people with higher risk of psychological distress—would be at higher risk. There has been vast research on mental health regarding hospital care workers during COVID-19 (22). However, the current literature has not identified individuals based on socio-economic factors, who are vulnerable to the pandemic across all occupations. Therefore, clearer results are needed to discern who needs social support.

Furthermore, research has only focused on some countries and has not covered worldwide issues, especially since COVID-19 did not continue to pose as serious a threat in several countries (23–26). During the Great Recession, Americans were more adversely affected than Europeans by the country’s lack of robust safety net programs, which mitigated the psychological impact of decrease in economic activity (27). Hence, more comprehensive analysis using various demographic and social factors with worldwide surveys is needed. This research was conducted to highlight demographic and social factors of a specific population group at high risk of psychological distress due to COVID-19. Herein, we aim to examine the hypothesis that decrease in economic activity due to COVID-19 is significantly associated with psychological distress, using obtained data from 15 countries in three regions, including Asia (China, Hong Kong-China, Indonesia, Malaysia, the Philippines, Singapore, South Korea, Taiwan, Thailand, Vietnam); Europe (Poland, Germany, Sweden, Turkey, Ukraine); and America (Canada, United States). We hope that our comprehensive analysis elucidates vulnerable populations and helps establish guidelines to address mental concerns in the aftermath of COVID-19.

## 2. Methods

### 2.1. Data set and participants

Online self-report questionnaires were administered in the 15 countries mentioned above. Most countries conducted survey using online survey website from the United States, except that Korea use their own survey website. The informed consent was obtained from each participant before the survey starts. The participants were selected by age-sex stratified random sampling with the goal of 1,000 people in each country. For an accurate survey on employment status, subjects aged above 20 and under 65 years old of working age were selected (Supplementary Figure S1). The exclusion criteria were as follows: participants who did not answer job loss or employment status questionnaires; participants who did not answer questions about psychological distress of depressive symptom or anxiety symptom; and participants who were missing their responses about each category including “sex,” “employment status,” “depressive symptom,” “anxiety symptom” of the questionnaires.

The study protocol was in accordance with the ethical guidelines of the 1975 Declaration of Helsinki and was approved by the Institutional Review Board of Severance Hospital (IRB: Y-2020-0088). All participants provided informed consent before completing questionnaires.

### 2.2. Definition and evaluation of data

#### 2.2.1. Psychological distress

The primary outcome variable of the study was psychological distress due to the COVID-19 pandemic. The participants were asked to indicate the extent to which they had feelings of depressive symptom and anxiety symptom, respectively. The question was: “How much have you been feeling the following emotions (depressed, anxious) during the pandemic, relative to how much you experienced them before the pandemic?” The responses were “less,” “same,” and “more.”

#### 2.2.2. Decrease in economic activity due to COVID-19

The “Decrease in economic activity due to COVID-19” group was defined based on one of the following criteria: (1) who responded “Reduced income” or “Loss of job” to the following question “Have you experienced any difficulties during the COVID-19 crisis?”; (2) those who lost a job or hours of work because their employer shut down or downsized due to COVID-19; and (3) who responded “Lost a job or hours because my employer shut down/downsized due to COVID-19” or “Left a job because I did not think it safe to work during the COVID-19 crisis.” to the following question “Which statement best describes your current employment status?”

#### 2.2.3. Education level

The participants were asked to indicate their highest level of education as follows: “Less than high school,” “High school,” “Some college or post-secondary education,”’ “4-year college graduate,” “Graduate or Professional training beyond college,” “Doctoral degree (PhD).” We recategorized the education level into 4 groups by combining the first and last two groups into one group. Consequently, the education level was classified into four categories: “High school or less,” “College,” “University,” and “Graduate or more.”

### 2.3. Statistical analysis

The differences in baseline characteristics stratified by gender were examined using chi-squared and t-tests for categorical and continuous variables. Prevalence of decrease in economic activity due to the COVID-19 pandemic was calculated according to country, gender, age, and education. Odds ratio (OR) with 95% confidence intervals (CIs) of psychological distress and decrease in economic activity due to COVID-19 pandemic was calculated using multiple logistic regression analysis. Several covariates such as country, gender, age, and education level were included in the multivariate logistic regression models. To find associating factors, we used a mixed model to regard age, gender, economic status as fixed effects, and country and education as random effects. A *p* value less than 0.05 was considered as statistically significant. All statistical analyses were performed using R version 4.0.2 (The R Foundation for Statistical Computing, Vienna, Austria).

## 3. Results

From a total of 16,942 participants, 2,298 either under 20 or over 65 years of age and 401 with missing values were excluded. Ultimately, 14,243 (men = 7,153 or 50.2%; women = 7,090 or 49.8%) were enrolled in this study. Mean (standard deviation) age of the entire sample was 40.67 (12.23) years old. Among all participants, 3,098 (12.75%) lost their job and 5,734 (40.26%) suffered from psychological distress. Regarding education level, 6,182 (40.40%) of the participants were university graduates, followed by 3,254 (33.85%) with a graduate degree or higher. Of the remaining participants, 2,787 (19.57%) completed high school or less, and 2,020 (14.18%) had completed college education. Detailed information about each country’s baseline characteristics is summarized in Supplementary Table S1.

**Figure 1:**
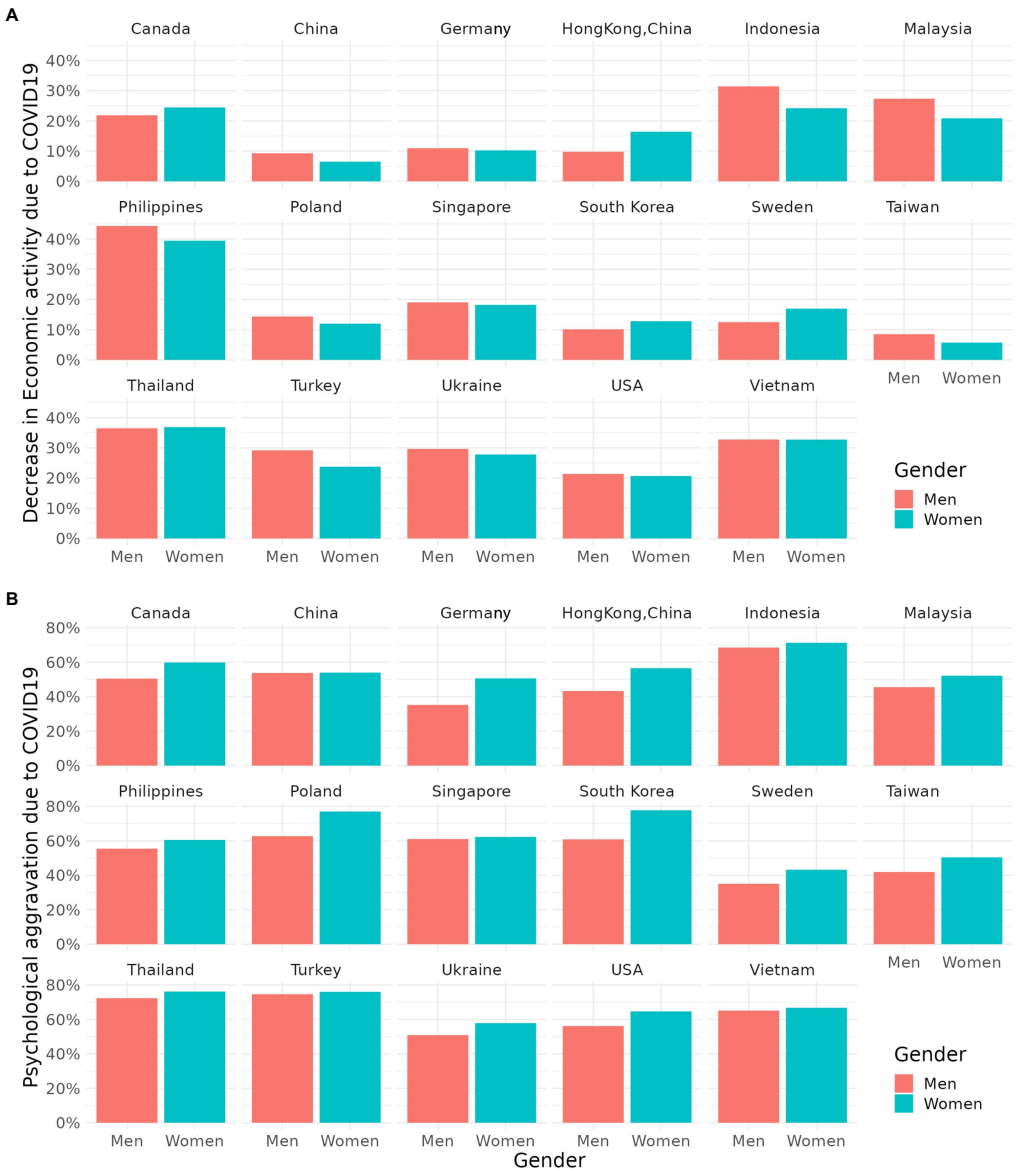
**(A,B)** Proportion of Job loss and psychological distress due to COVID-19 stratified by gender.

The proportions of decrease in economic activity and psychological distress due to COVID-19 stratified by gender in 15 countries are summarized in Figure 1. The average decrease in economic activity proportion of all countries was 21.75% (20.58% for women and 22.91% for their men counterparts). The top three countries with the highest unemployment rates due to COVID-19 were the Philippines (41.94%), Thailand (36.68%), and Vietnam (32.74%). Hong Kong was the country with the biggest difference in unemployment rates, which was higher for women than men (*p* = 0.033). Meanwhile in Indonesia (*p* = 0.014) and Malaysia (*p* = 0.032), men had higher unemployment rates than women.

However, women experienced more depressive symptom and anxiety symptom due to COVID-19 compared to men in all countries examined. The average prevalence of psychological distress in all countries was 59.74% (63.17% for women and 56.34% for men). The four countries which showed the largest difference in psychological distress by gender were South Korea (16.88%, < 0.001), Germany (15.42%, < 0.001), Poland (14.31%, < 0.001), and Hong Kong (13.21%, 0.003).

**Figure 2:**
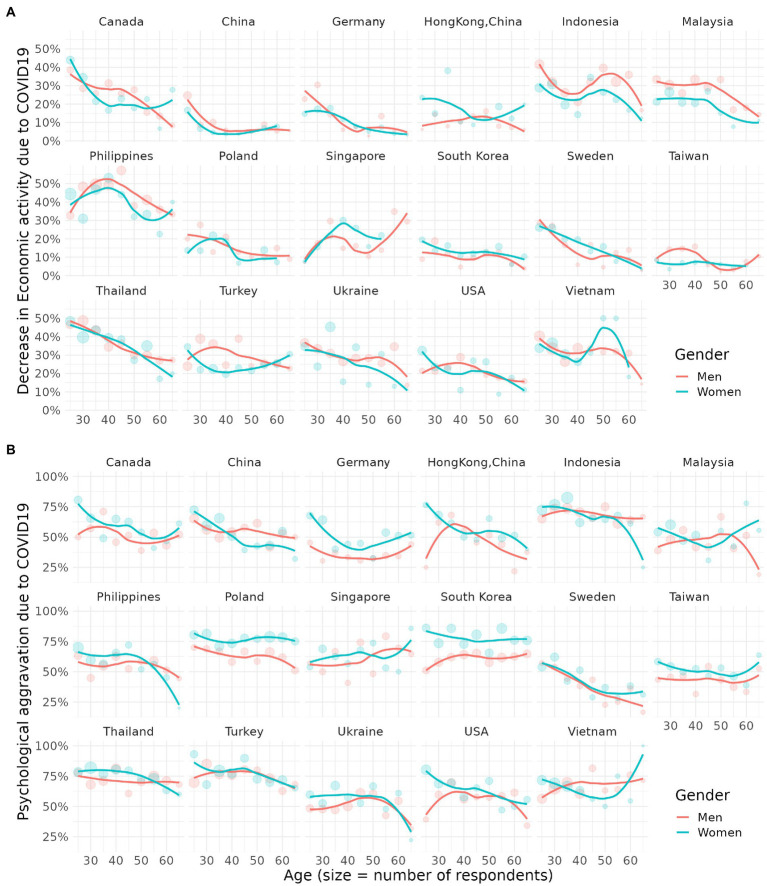
**(A,B)** Proportion of job loss and psychological distress due to COVID-19 stratified by age and gender.

Figure 2 illustrates the proportions of decrease in economic activity and psychological distress due to COVID-19 by country and age. In most countries, decrease in economic activity has been more frequent among younger demographic groups. It was especially prominent in Canada, Sweden, Thailand, and Ukraine. Singapore is one of the only countries that seemed to have higher decrease in economic activities for older people.

Moreover, young participants tend to feel more psychological distress due to COVID-19 compared to older ones, although this was not as prominent as decrease in economic activity. This was especially noticeable in Sweden, the Philippines, and China. However, Singapore and Vietnam showed the opposite result, while South Korea had a high rate of depressive symptom and anxiety symptom regardless of age.

**Figure 3:**
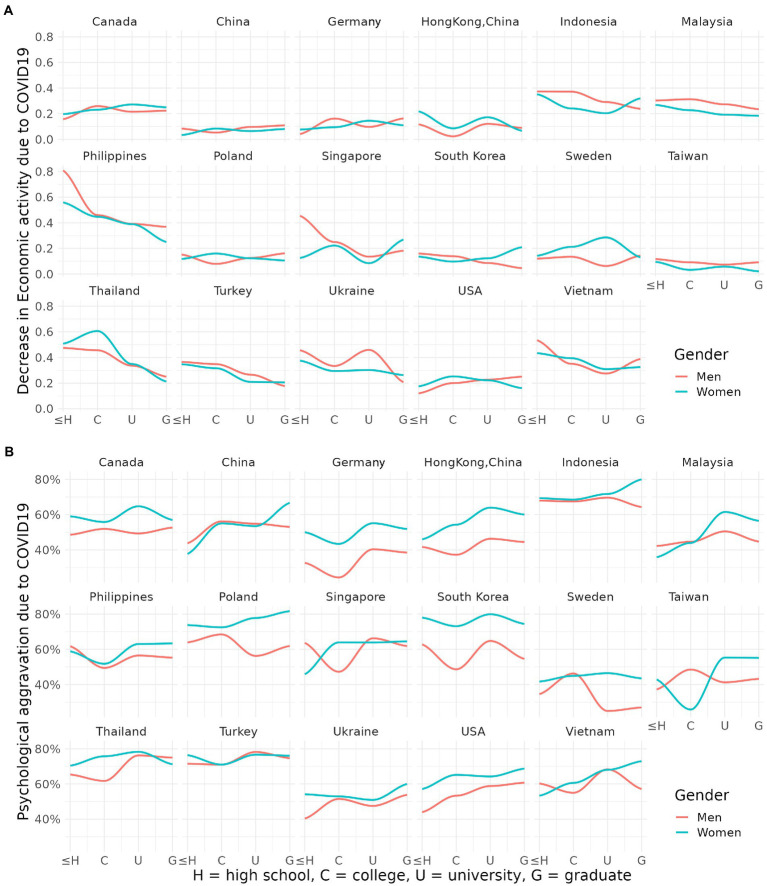
**(A,B)** Proportion of job loss and psychological distress due to COVID 19 analyzed by education level.

Decrease in economic activity was more common among adults with lower education levels and those without a university degree compared to those who graduated university or held a higher degree (Figure 3). This tendency stands out in the Philippines, Thailand, Vietnam, and Turkey. We hypothesized that people with lower education level would feel more depressive symptom and anxiety symptom. However, the results indicated otherwise—countries such as China, Malaysia, and the United States, which have a lower correlation between education and decrease in economic activity, also showed higher psychological distress with higher education level. Psychological distress rate in the USA with education level of high school or less, college, university, and graduate or more was 51.33, 6.053, 61.63, and 56.7%, respectively.

**Figure 4:**
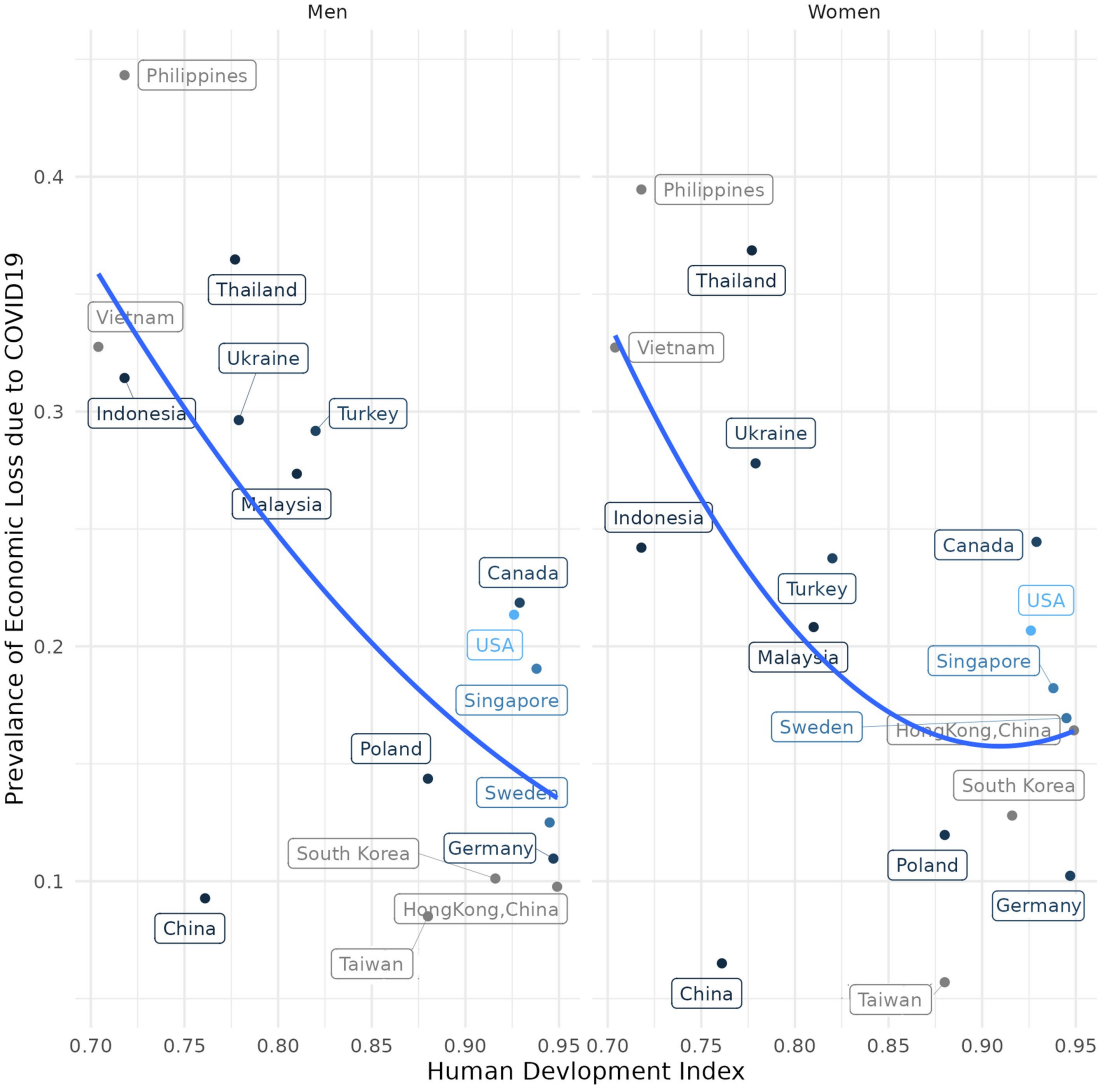
Human development Index versus job loss due to COVID-19 stratified by gender.

Figure 4 illustrates the prevalence of decrease in economic activity due to COVID-19 versus the Human Development Index (HDI) of each country stratified by gender. The HDI used in this study is from the 2020 UN Human Development Report, which is a statistical index composed of life expectancy, education, and *per capita* income indicators (28). HDI and proportion of decrease in economic activity due to COVID-19 were negatively associated both for men and women. Countries with lower HDI indexes indicated higher decrease in economic activity due to the COVID-19 pandemic. Moreover, for women, decrease in economic activity was lower in countries with higher HDI.

**Figure 5:**
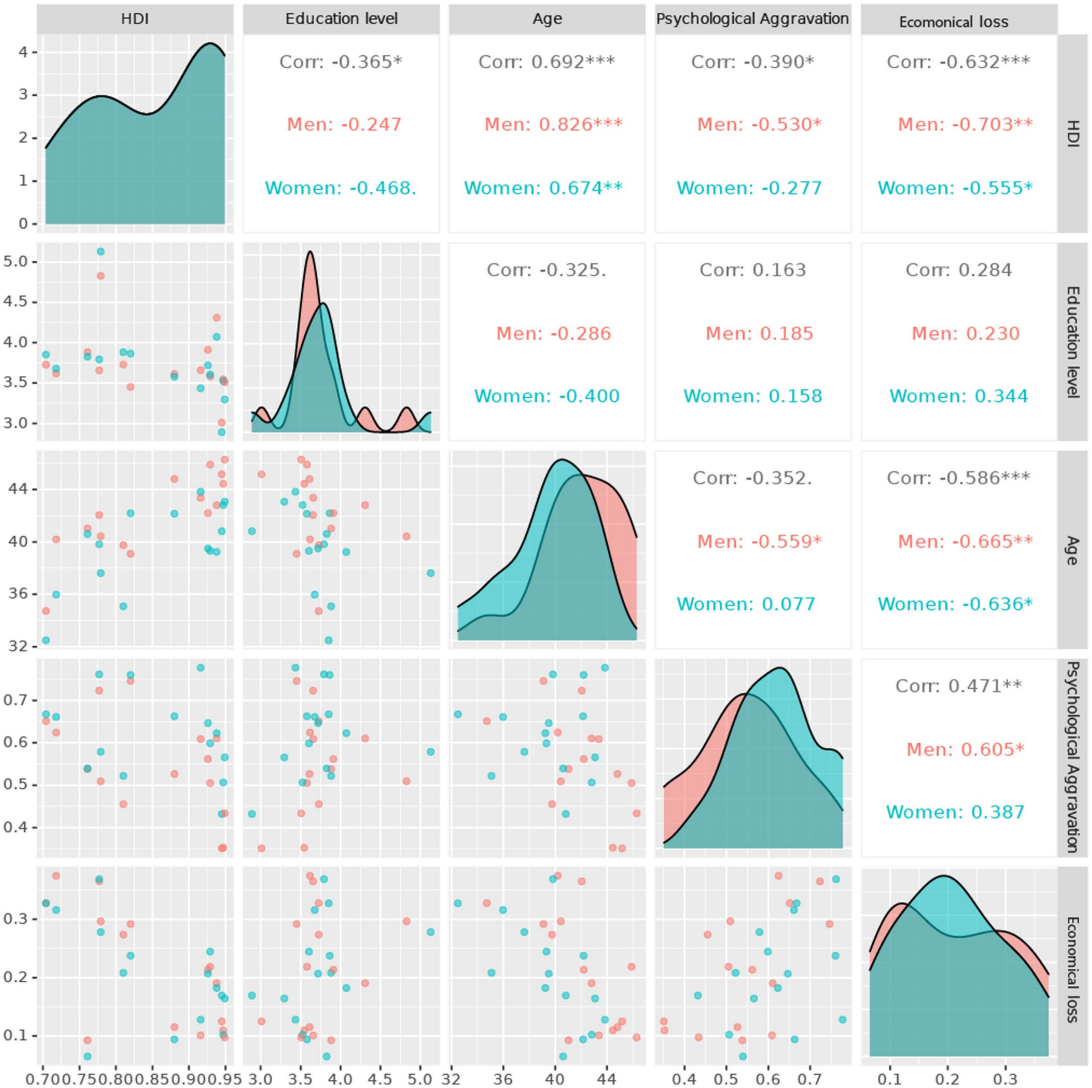
Correlation analysis plot of study variables.

Finally, Figure 5 shows analyzation of various variables such as HDI, education level, age, psychological distress, decrease in economic activity, by each sex strata. Age, HDI, decrease in economic activity presented stronger linear correlation among each other, with prominent extent of association in men.

The result of the logistic regression analysis (as OR and 95% CIs) of the association of psychological distress and decrease in economic activity, age, and gender stratified by HDI is shown in Table 1. As this was a mixed model, country and education level were regarded as random factors. For all countries, the ORs (95% CIs) for the depressive or anxious symptoms by decrease in economic activity were 1.101 (1.08–1.123) for economic status loss group, 0.998 (0.997–0.999) for age, and 1.067 (1.051–1.084) for women. Stratified with countries’ HDI levels, each group shows the same ORs for psychological distress as 0.998. The associations between women and decrease in economic activity were slightly higher in high HDI level countries as ORs (95% CIs) were 1.108 (1.078–1.138) and 1.137 (1.096–1.180) respectively, than low HDI level countries which were 1.029 (1.007–1.051) and 1.084 (1.058–1.111).

**Table 1:** Associations between psychological distress and HDI, economic status, age, gender.

HDI	Predictors		OR	LL	UL
All	*Economic status*	*Active*	*1*	*(Reference)*	
		*Loss*	1.101	1.08	1.123
	Age		0.998	0.997	0.999
	Gender	Men	*1*	*(Reference)*	
		Women	1.067	1.051	1.084
>0.9	*Economic status*	*Active*	*1*	*(Reference)*	
		*Loss*	1.137	1.096	1.180
	Age		0.998	0.996	0.999
	Gender	Men	*1*	*(Reference)*	
		Women	1.108	1.078	1.138
>0.8	*Economic status*	*Active*	*1*	*(Reference)*	
		*Loss*	1.086	1.042	1.133
	Age		0.998	0.997	1
	Gender	Men	*1*	*(Reference)*	
		Women	1.075	1.042	1.11
<0.8	*Economic status*	*Active*	*1*	*(Reference)*	
		*Loss*	1.084	1.058	1.111
	Age		0.998	0.997	0.999
	Gender	Men	*1*	*(Reference)*	
		Women	1.029	1.007	1.051

Mixed Model: country and education are regarded as random effect.

## 4. Discussion

COVID-19 has caused an economic crisis and numerous job losses worldwide (29, 30). This study investigated the associations among decrease in economic activity, gender, and psychological distress with consideration of economic status of countries and education using multinational surveys. The results revealed that women had a higher prevalence of psychological distress than men, and younger age was significantly associated with decrease in economic activity. Moreover, countries with lower HDI showed a higher prevalence of decrease in economic activity, especially at lower education levels. Psychological distress due to COVID-19 shows significant associations with decrease in economic activity, women, and younger age. While the proportion of decrease in economic activity was different for each country, the degree of association of the individual factors was the same. These results show consistency with other studies, especially showing exacerbation in women. Also, some studies have shown that specific nation’s demographic groups suffered disparities in the workforce, according to skin color and immigrant status (31, 32). In these 15 countries, the association between gender and unemployment rate varies from country to country. In Hong Kong, where women have a significant unemployment rate relative to men, the ratio of female to male labor force participation rate was 79.58% in 2019. In Malaysia (2020) and Indonesia (2021), which shows significant higher decrease in economic activity for men, the ratio of female to male labor force participation rate was 68.6 and 65.3%, respectively (33). Therefore, this gender difference in decrease in economic activity may be due to differences in labor force participation. Moreover, Dang and Nguyen announced that women expected their labor income to fall by 50% more than men, which might affect psychological distress (31). Gender difference in the participation rate can be attributed to the fact that women were more likely to work in fields which required face-to-face contact. Thus, this situation may have been impacted by social distancing guidelines (31, 34).

Decrease in economic activity was also related to younger age and psychological distress. This result corresponds to research stating that the COVID-19 recession hit young workers the hardest since February 2020 in the United States (35). Moreover, Major et al. reported that those between 16 and 25 years were twice as likely as older employees to have suffered decrease in economic activity due to the COVID-19 (36). Several explanations were offered. For instance, younger employees may have been predominantly employed in industries that were the hardest hit, such as service and sales, hospitality and leisure, and retail trade (14). Therefore, higher decrease in economic activities in younger workers compared to older counterparts may account for the participation rate gap in the retail trade, leisure, and hospitality industry (37). Furthermore, young workers have been excluded from certain COVID-19 assistance in several countries (37).

Education level and decrease in economic activity were negatively correlated in some countries with low HDI. This supports the idea that the labor market disruptions have affected workers in a wide set of industries and occupations, and those without a college degree experienced the most severe impacts (38). Addressing gaps in educational attainment might be important to create better economic resiliency for individuals against future emergent circumstances.

Even though decrease in economic activity in low education level participants was higher, countries such as the Philippines, Thailand, and Turkey did not show distinct psychological distress difference between education groups. A possible explanation can be made that this reflects a “steeling effect” among lower HDI populations with lower education levels. They have probably experienced past adversity, and therefore, acquired more resilience for dealing with sudden negative experiences (39).

HDI and prevalence of decrease in economic activity due to COVID-19 have a negative association in both men and women. Lee and Chang examined the relationship between the tourism sector and growth of the economy (40). The result affirmed that tourism development has a greater impact on gross domestic product (GDP) in non-Organization for Economic Cooperation and Development (OECD) countries than in OECD countries, which implies that countries dependent on tourism with lower HDI might be affected more by the COVID-19 recession. Moreover, individuals working in tourism industries show relatively low education levels compared with workers in general (41).

Furthermore, the proportion of travel and tourism industry field among the global GDP decreased to 5.5% in 2020 from 10.4% of 2019 (42). This decline over the previous year was because of the COVID-19 pandemic which disrupted worldwide travel (42). Therefore, we reason that COVID-19 worsened the economic situation of low HDI-scoring countries by impacting the tourism industry and thereby the increasing the decrease in economic activity of workers at a lower education level.

Unemployment disproportionately affects the economically vulnerable, raising concerns about worsening social inequality. Given the high prevalence of unemployment among women and young workers, there is an urgent need to improve the availability and affordability of mental health services, as well as the need for financial aid and job creation programs for them. Psychological interventions and economical support alone cannot directly solve the underlying problem of decrease in economic activity; however, they might help an individual stay confident and motivated to persevere with job searching when the economy rebounds. In addition, it might be possible to provide not only wage compensation, but also skills training or job relocation. Such measures could help rebuild the careers of lower education workers in low HDI countries, especially in the tourism industry (43).

## 5. Strength

There are several strengths in our study. The COVID-19 pandemic provides a unique opportunity for analyzing the implications of unemployment for mental health as the cause of decrease in economic activity is exogenous to individuals. In addition, decrease in economic activity related to national lockdown reflects characteristics such as national countermeasures, outbreaks, and economic structure under the same conditions between countries, which provide the settings for a natural experiment. Studies on the COVID-19 scenario have usually focused on macroeconomic statuses such as GDP. In this study, individual psychological factors were directly related with decrease in economic activity. Unlike previous research that has limited data from limited countries and regions, this research is one of the few papers that looked at the psychological problem of COVID at the multinational level. A total of 15 countries participated in this study, which facilitated comparisons between countries with a large sample size (*n* = 14,243). Therefore, this study might be used as a cornerstone to set policies for the vulnerables who need financial support and psychological intervention.

## 6. Limitation

However, this study has several limitations. First, as it used a cross-sectional, correlational design, this study cannot be used to clarify whether decrease in economic activity precedes the psychological symptom or occurs as a result of certain behaviors by worker who are already depressed or anxious. Therefore, additional longitudinal investigations are needed. Second, there is a lack of information about confounding factors, which may be related to psychological symptoms other than decrease in economic activity. For instance, study participants may have a history of depression, anxiety disorder, sleep disturbance, and drug use. Moreover, social distancing due to COVID-19 might affect the mental health of study participants directly; this issue should be considered in future studies. Another drawback is that the reliability of data obtained through online self-information research is not guaranteed. Finally, as the defining psychological symptom was based on one question, it does not ensure questionnaire validity and is less sensitive to variation in, or the severity of, psychological symptoms as opposed to more comprehensive tools such as the Patient Health Questionnaire-9 (PHQ-9) and the Center for Epidemiological Studies-Depression Scale (CES-D) 10. As cross-sectional study correlation between SAR-CoV-2 variants and COVID-19 variants cannot be described.

## 7. Conclusion

Our study showed that decrease in economic activity, younger age, and being women were significantly associated with psychological distress. Moreover, women in higher HDI countries and lower education levels in lower HDI countries were considered vulnerable. Therefore, improved policies and guidelines for relevant financial aid and psychological intervention are needed, especially for the vulnerable populations.

## Data availability statement

The original contributions presented in the study are included in the article/Supplementary material, further inquiries can be directed to the corresponding authors.

## Author contributions

MK: conceptualization, resources, data curation, formal analysis, investigation, visualization, methodology, writing—original draft. BY: conceptualization, investigation, writing—review and editing. JS, AC, and JO: funding acquisition, project administration. JK: data curation. KN: investigation, resources. LR: supervision, investigation, resources, validation. J-HY: supervision, resources, investigation, validation, writing—review and editing. All authors contributed to the article and approved the submitted version.

## Funding

This work was supported under the framework of international cooperation program managed by the National Research Foundation of Korea (NRF-2021K2A9A1A01102239).

## Conflict of interest

The authors declare that the research was conducted in the absence of any commercial or financial relationships that could be construed as a potential conflict of interest.

## Publisher’s note

All claims expressed in this article are solely those of the authors and do not necessarily represent those of their affiliated organizations, or those of the publisher, the editors and the reviewers. Any product that may be evaluated in this article, or claim that may be made by its manufacturer, is not guaranteed or endorsed by the publisher.
