# Supplementary figures and images for: Risk of psychological distress by decrease in economic activity, gender, and age due to COVID-19: A multinational study
Source: Front Public Health. 2023 May 10;11:1056768. doi: 10.3389/fpubh.2023.1056768 (PMC10208271; doi:10.3389/fpubh.2023.1056768)

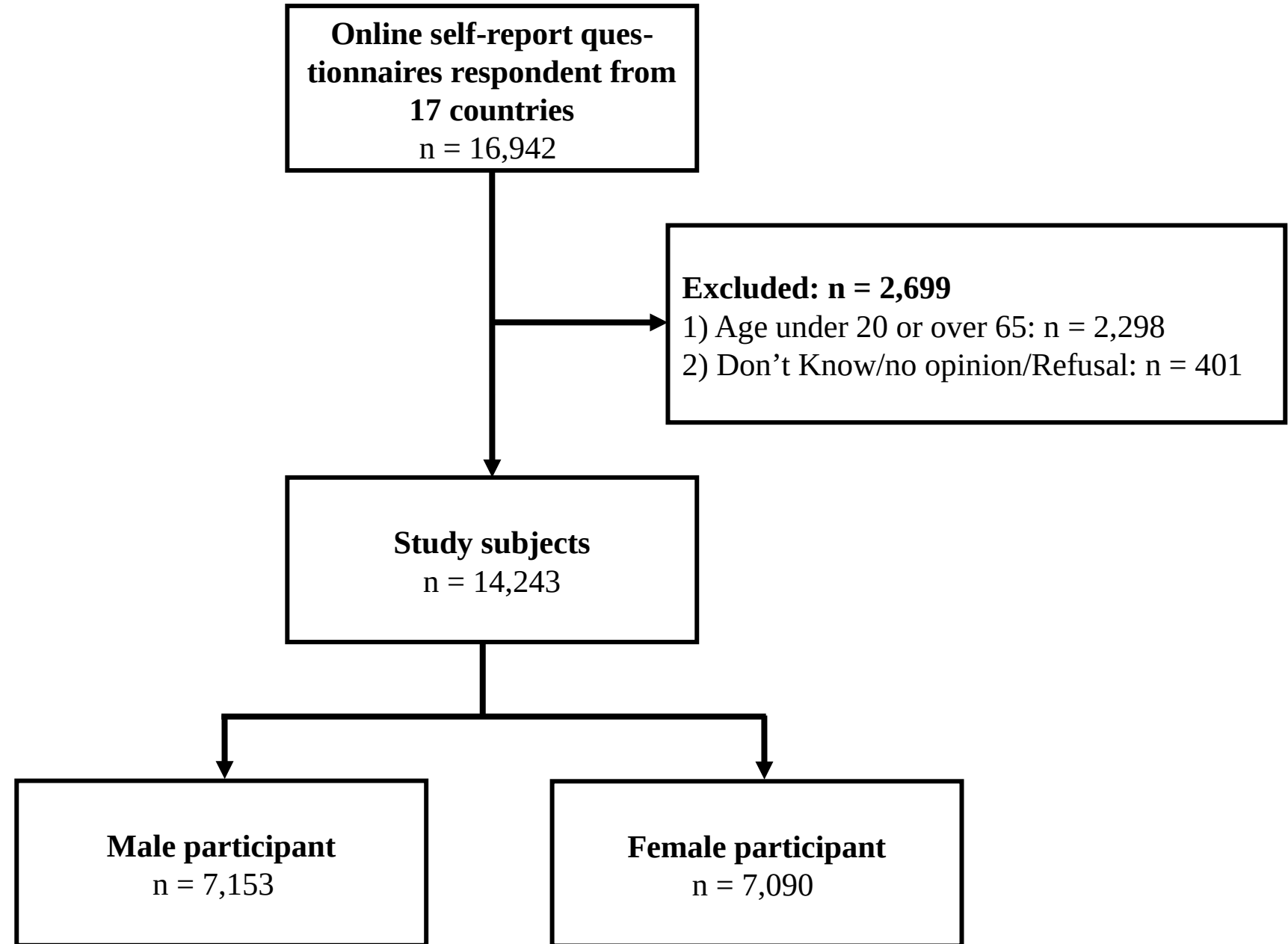

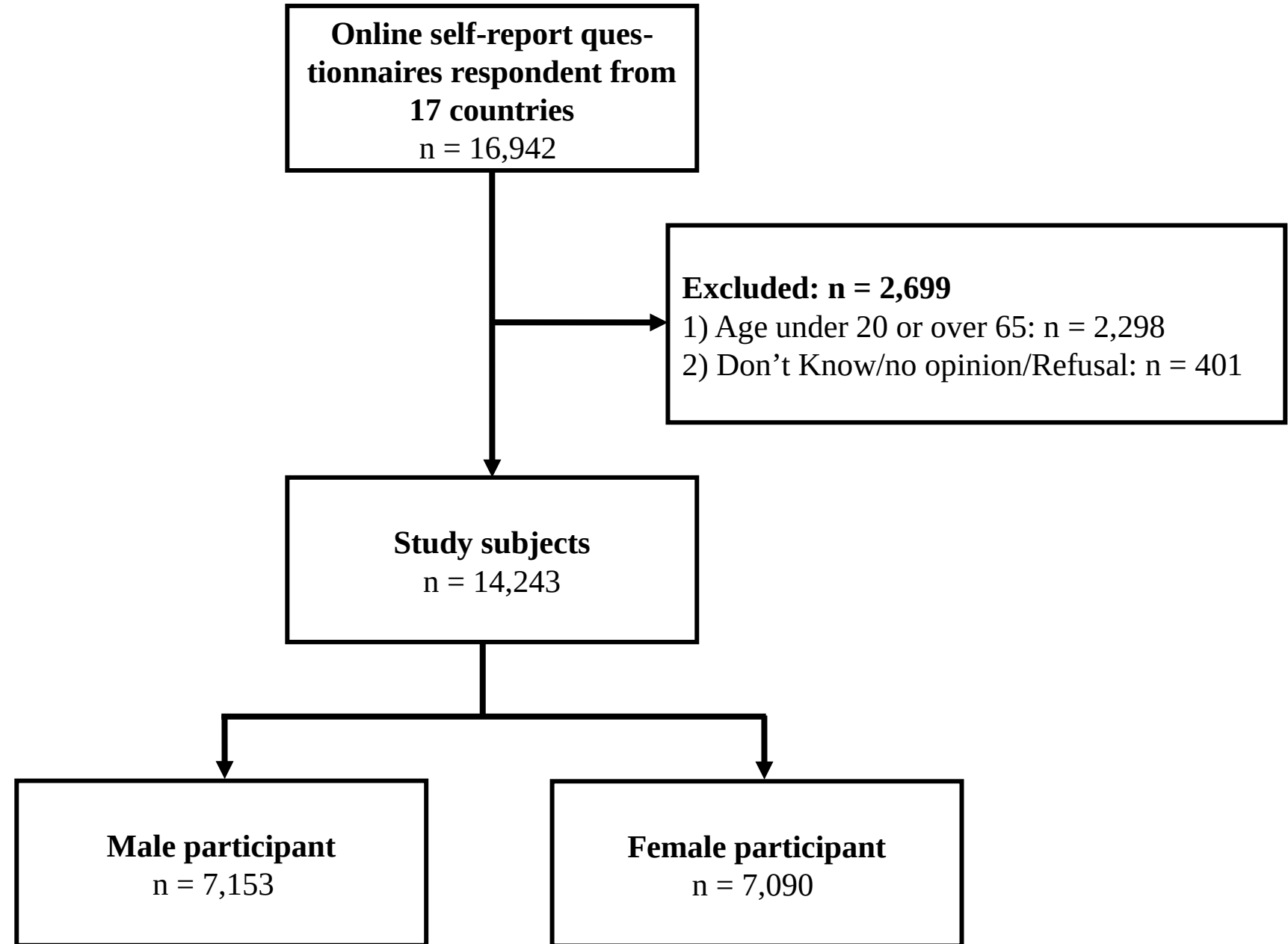

Supplement: Supplementary file 1 [file Data_Sheet_1.PDF]
