# Supplementary material for: Risk of psychological distress by decrease in economic activity, gender, and age due to COVID-19: A multinational study
Source: Front Public Health. 2023 May 10;11:1056768. doi: 10.3389/fpubh.2023.1056768 (PMC10208271; doi:10.3389/fpubh.2023.1056768)
Supplement: Supplementary file 2 [file Table_1.DOCX]

| **Supplementary Table S1. Baseline characteristics of participants by country** | | | | | |  |  |  |  |
| --- | --- | --- | --- | --- | --- | --- | --- | --- | --- |
| **Variable** | **Canada** | **China** | **German** | **HongKong,**  **China** | **Indonesia** | **Malaysia** | **Philippines** | **Poland** | **Singapore** |
| **Decrease in economic activity** |  |  |  |  |  |  |  |  |  |
| No | 586  (76.90%) | 1080  (92.07%) | 619  (89.45%) | 460  (86.79%) | 717  (71.99%) | 656  (75.58%) | 522  (58.06%) | 766  (87.05%) | 380  (81.37%) |
| Yes | 176 (23.10%) | 93 (7.93%) | 73 (10.55%) | 70 (13.21%) | 279 (28.01%) | 212 (24.42%) | 377 (41.94%) | 114 (12.95%) | 87 (18.63%) |
|  |  |  |  |  |  |  |  |  |  |
| **Psychological Aggravation** |  |  |  |  |  |  |  |  |  |
| No | 343 (45.01%) | 541 (46.12%) | 388 (56.07%) | 264 (49.81%) | 300 (30.12%) | 447 (51.50%) | 378 (42.05%) | 254 (28.86%) | 179 (38.33%) |
| Yes | 419 (54.99%) | 632 (53.88%) | 304 (43.93%) | 266 (50.19%) | 696 (69.88%) | 421 (48.50%) | 521 (57.95%) | 626 (71.14%) | 288 (61.67%) |
|  |  |  |  |  |  |  |  |  |  |
| **Age** |  |  |  |  |  |  |  |  |  |
|  | 42.76  (12.60) | 40.83 (12.17) | 43.53  (12.28) | 44.62  (11.82) | 38.13  (11.28) | 37.66  (11.31) | 38.20  (12.00) | 44.03  (12.40) | 41.00  (11.64) |
|  |  |  |  |  |  |  |  |  |  |
| **Gender** |  |  |  |  |  |  |  |  |  |
| Men | 398 (52.23%) | 604 (51.49%) | 301  (43.50%) | 256 (48.30%) | 525 (52.71%) | 479 (55.18%) | 458 (50.95%) | 362 (41.14%) | 231 (49.46%) |
| Women | 364 (47.77%) | 569 (48.51%) | 391 (56.50%) | 274 (51.70%) | 471 (47.29%) | 389 (44.82%) | 441 (49.05%) | 518 (58.86%) | 236 (50.54%) |
|  |  |  |  |  |  |  |  |  |  |
| **Education level** |  |  |  |  |  |  |  |  |  |
| High school or less | 131 (17.19%) | 109 (9.29%) | 230 (33.24%) | 147 (27.74%) | 222 (22.29%) | 176 (20.28%) | 81 (9.01%) | 337 (38.30%) | 35 (7.49%) |
| college | 203 (26.64%) | 117 (9.97%) | 90 (13.01%) | 78 (14.72%) | 97 (9.74%) | 149 (17.17%) | 172 (19.13%) | 107 (12.16%) | 72 (15.42%) |
| University | 280 (36.75%) | 777 (66.24%) | 131 (18.93%) | 230 (43.40%) | 610 (61.24%) | 226 (26.04%) | 510 (56.73%) | 138 (15.68%) | 157 (33.62%) |

| **Variable(continued)** | **South Korea** | **Sweden** | **Taiwan,**  **China** | **Thailand** | **Turkey** | **Ukraine** | **USA** | **Vietnam** | **Overall** | **p-value** |
| --- | --- | --- | --- | --- | --- | --- | --- | --- | --- | --- |
| **Decrease in economic activity** |  |  |  |  |  |  |  |  |  | <0.001 |
| No | 914  (88.57%) | 600  (84.87%) | 643  (92.92%) | 644  (63.32%) | 701  (73.56%) | 639  (71.08%) | 610  (79.02%) | 608  (67.26%) | 11145 (87.75) |  |
| Yes | 118 (11.43%) | 107 (15.13%) | 49 (7.08%) | 373 (36.68%) | 252 (26.44%) | 260 (28.92%) | 162 (20.98%) | 296 (32.74%) | 3098 (12.75%) |  |
|  |  |  |  |  |  |  |  |  |  |  |
| **Psychological Aggravation** |  |  |  |  |  |  |  |  |  | <0.001 |
| No | 318 (30.81%) | 425 (60.11%) | 372 (53.76%) | 261 (25.66%) | 235 (24.66%) | 417 (46.38%) | 303 (39.25%) | 309 (34.18%) | 5734 (40.26%) |  |
| Yes | 714 (69.19%) | 282 (39.89%) | 320 (46.24%) | 756 (74.34%) | 718 (75.34%) | 482 (53.62%) | 469 (60.75%) | 595 (65.82%) | 8509 (59.74%) |  |
|  |  |  |  |  |  |  |  |  |  |  |
| **Age** |  |  |  |  |  |  |  |  |  | <0.001 |
|  | 43.60  (12.30) | 42.59  (12.71) | 42.46  (11.86) | 40.89  (12.10) | 40.65  (12.26) | 39.34  (11.40) | 40.74  (12.88) | 33.78  ( 9.51) | 40.67 (12.23) |  |
|  |  |  |  |  |  |  |  |  |  |  |
| **Gender** |  |  |  |  |  |  |  |  |  | <0.001 |
| Men | 524 (50.78%) | 288 (40.74%) | 341 (49.28%) | 488 (47.98%) | 473 (49.63%) | 550 (61.18%) | 356 (46.11%) | 519 (57.41%) | 7153 (50.22%) |  |
| Women | 508 (49.22%) | 419 (59.26%) | 351 (50.72%) | 529 (52.02%) | 480 (50.37%) | 349 (38.82%) | 416 (53.89%) | 385 (42.59%) | 7090 (49.78%) |  |
|  |  |  |  |  |  |  |  |  |  |  |
| **Education level** |  |  |  |  |  |  |  |  |  | <0.001 |
| High school or less | 212 (20.54%) | 346 (48.94%) | 106 (15.32%) | 149 (14.65%) | 209 (21.93%) | 81 (9.01%) | 113 (14.64%) | 103 (11.39%) | 2787 (19.57%) |  |
| college | 165 (15.99%) | 185 (26.17%) | 64 (9.25%) | 101 (9.93%) | 107 (11.23%) | 50 (5.56%) | 190 (24.61%) | 73 (8.08%) | 2020 (14.18%) |  |
| University | 546 (52.91%) | 44 (6.22%) | 385 (55.64%) | 645 (63.42%) | 441 (46.27%) | 175 (19.47%) | 245 (31.74%) | 642 (71.02%) | 6182 (43.40%) |  |
| Graduate or more | 109 (10.56%) | 132 (18.67%) | 137 (19.80%) | 122 (12.00%) | 196 (20.57%) | 593 (65.96%) | 224 (29.02%) | 86 (9.51%) | 3254 (33.85%) |  |
| Values are expressed by n (%) or mean (SD). | |  |  |  |  |  |  |  |  |  |
